# Barriers to Undergoing Body-Contouring Surgery Following Bariatric Surgery in Saudi Arabia

**DOI:** 10.7759/cureus.50558

**Published:** 2023-12-15

**Authors:** Tareq Alyahya, Mohammed A Albesher, Haidar A Alessa, Zahra B Alali, Abdulrahman T Al-Mulla

**Affiliations:** 1 Plastic Surgery, King Faisal University, Al-Ahsa, SAU; 2 Medicine, King Faisal University, Al-Ahsa, SAU

**Keywords:** bariatric surgery, abdominoplasty, body lift, excess sagging, body-contouring surgery

## Abstract

Introduction

The prevalence of obesity has experienced a significant global increase in recent years, emerging as a prominent worry affecting numerous individuals throughout various countries, including Saudi Arabia. Bariatric surgery, a common treatment, often leads to excess skin. Despite its benefits, few patients choose body contouring surgery. A cross-sectional study aims to identify barriers, including socioeconomic and psychological factors.

Methodology

This is a cross-sectional study conducted in Saudi Arabia. Participants included those who underwent bariatric surgery. Data were collected through questionnaires and analyzed by Statistical Product and Service Solutions (SPSS, version 29) (IBM SPSS Statistics for Windows, Armonk, NY).

Results

Our study involved 662 Saudi participants with post-bariatric surgery, primarily females (386, 58.3%), aged 19-29 (44.3%). Most had undergone bariatric surgery (558, 84.3%), mainly gastric sleeve (485, 73.3%). Excess skin was a common issue (311, 47.0%). Difficulties included rashes and emotional distress (e.g., depression). About 8.3% had body-contouring surgery, including body lifts (13, 23.6%) and liposuction (19, 34.5%). Factors influencing surgery decisions included self-confidence (123, 18.6%) and cost (9.9%). Barriers for 32.2% considering surgery included cost (80.2%) and fear of a second surgery (45.6%). Females (67.1%), Saudis (85.4%), and employed individuals (49.3%) were more likely to consider surgery (p < 0.05).

Conclusion

Our study highlights the complexity of body-contouring decisions after bariatric surgery in Saudi Arabia. Cost and fear were barriers; females, Saudis, and employed individuals were more likely to consider surgery. A patient-centered approach, addressing barriers, and offering support are crucial for informed choices and improved well-being.

## Introduction

Weight gain that is abnormal or excessive and poses a risk to health is what is meant by the terms "overweight" and "obesity." Overweight is defined as a body mass index (BMI) of 25, and obesity is a BMI greater than 30. Since 1975, the global rate of obesity has nearly tripled according to the WHO. According to a recent study, the prevalence of obesity was 35.6% in Saudi Arabia [1]. A BMI of 40 kg/m^2^ with or without an obesity-related comorbidity or 35 kg/m^2^ in the presence of an obesity-associated illness is stated as an indication for bariatric surgery in both the Saudi Clinical Practice Guidelines for the Management of Obesity and the SA Society for Metabolic and Bariatric Surgery, which are the same indications as in the United States [2,3]. Bariatric surgery is a procedure used to assist obese patients in losing weight. Bariatric surgery comes in various forms, and each one alters how the digestive system functions. Some varieties reduce the capacity of the stomach, which causes a person to feel full more quickly and eat less food [4]. Since 2014, bariatric surgery has remained the most frequently performed elective procedure [5]. In 2019, there were approximately 27,000 bariatric procedures in Saudi Arabia [6]. This procedure is quite helpful in treating many other conditions in addition to obesity, including diabetes, high blood pressure, sleep apnea, and high cholesterol [7]. However, with this rapid weight loss, most patients will experience loosening of the skin, which is unwanted by most patients. Regardless of their age or gender, most post-bariatric patients report having excess skin in various body areas. Most post-bariatric patients have physical, functional, and emotional limitations caused by excess skin. As a result, abdominal contouring surgery is the most requested procedure after bariatric surgery [8]. Body contouring surgeries aim to remove the excess sagging skin and fat, resulting in an overall better shape and general appearance. Some of the body contouring procedures are body lift and abdominoplasty, which involve removing the extra skin that hangs over the abdomen and are the most performed contouring procedures. Others include arm lift, breast lift, and thigh lift, which follow the same principle [9]. A study regarding patients' long-term satisfaction after plastic surgery following gastric bypass showed that a greater number of patients had a much better quality of life in almost all subdomains: self-esteem, social life, workability, sexual activity, and physical activity [10]. Another qualitative study done in the USA also concluded the importance of body contouring surgery in enhancing physical, psychological, and social well-being [11]. Despite all that has been mentioned, there are still a few people undergoing contouring surgery after bariatric surgery. According to a study done in Saudi Arabia, which involved 128 patients who underwent bariatric surgery, 78.1% of patients expressed a desire for body contouring surgery. Only 18 patients (14%) have undergone body contouring surgery [12]. Another study done on adolescents shows that only 25 (12.6%) of the 198 study participants for whom BCS information was available received 41 body contouring surgeries following bariatric surgery [13]. As a result, there is a need for a study to be conducted on the barriers that could prevent people who underwent bariatric surgery from getting body contouring surgery. A cross-sectional study was conducted to explore the factors that determine patients' decision whether to have body contouring surgery in association with their socioeconomic variables and the psychological aspects as well.

## Materials and methods

Study methodology

A cross-sectional study was conducted over a period of six months after approval from the ethical committee. The study was conducted among a population of females and males aged 16 years and above. The data were collected using a purposive sampling technique.

Study population

This study was carried out among the female and male population who had undergone bariatric surgery in Saudi Arabia.

Sample size

The total number of participants in our sample was at least 300 in Saudi Arabia. It was calculated using the formula (n = z^2 * P * (p-1) / e^2). Here, N represents the sample size, z is the confidence level at 95%, P is the expected true proportion (0.5), and e is the margin of error (0.05).

Sample technique

A simple random sample was used.

Inclusion criteria

Females and males who had undergone bariatric surgery and were aged 16 and above were included. Subjects were required to be residents of Saudi Arabia.

Exclusion criteria

Individuals below the age of 16 and those who were not residents of Saudi Arabia were excluded.

Study procedure

Participants who fulfilled the inclusion and exclusion criteria and provided consent were enrolled. Each subject anonymously filled out the questionnaire. The results were statistically analyzed using Statistical Product and Service Solutions (SPSS, version 29) (IBM SPSS Statistics for Windows, Armonk, NY). The study timeline was six months from obtaining the necessary approvals. The data collected from the IBM SPSS program were interpreted accordingly. Interpretation of the collected data was performed, and potential solutions were proposed when applicable.

Data management

The data were stored in a secure location, and only approved personnel had access to the data. Privacy and confidentiality were prioritized, and the study ensured that participants' identities remained anonymous. The data were analyzed using the IBM SPSS program and interpreted by the investigator.

Statistical analysis

Data analysis was performed using SPSS, version 29. Categorical variables were presented using frequency and percentages. Numerical variables were summarized using minimum, maximum, mean, and standard deviation. The chi-square test was used to compare variables. The significance level was set at 0.05.

## Results

Our study included 662 participants in Saudi Arabia considering body-contouring surgery after bariatric surgery. Table 1 shows that the majority were female (386, 58.3%), aged 19-29 (44.3%), overweight 195(29.5%), and Saudi nationals (606, 91.5%). About 50% were married, and 71.8% had a university-level education. Most participants were employed (329, 49.7%), and a significant portion had a monthly income below 2500 SAR (262, 39.6%).

**Table 1:** Sociodemographic Features of the Participants

		Frequency (n=662)	Percent
Gender	Female	386	58.3
	Male	276	41.7
Age	< 18 Years	19	2.9
	19-29 Years	293	44.3
	30-39 Years	169	25.5
	40-49 Years	133	20.1
	50-59 Years	41	6.2
	60 or Greater	6	0.9
BMI	Underweight	13	2.0
	Normal	136	20.5
	Overweight	195	29.5
	Class 1 Obese	112	16.9
	Class 2 Obese	71	10.7
	Class 3 Obese	119	18.0
Nationality	Non-Saudi	56	8.5
	Saudi	606	91.5
Marital status	Single (Never Married)	294	44.4
	Married	331	50.0
	Divorced	28	4.2
	Widowed	9	1.4
Educational level	Basic education (up to secondary level)	155	23.4
	University level	475	71.8
	Non-university	32	4.8
Occupation	Student	152	23.0
	Employed	329	49.7
	Unemployed	78	11.8
	Housewife	103	15.6
Monthly income	< 2500 SAR	262	39.6
	2500-4999 SAR	118	17.8
	5000-9999 SAR	144	21.8
	10000-19999 SAR	98	14.8
	> 20,000 SAR	40	6.0

Table 2 shows the features of participants related to bariatric surgery. Most had undergone bariatric surgery (558, 84.3%), with a significant weight reduction from 121.1 to 84.7 kg. Gastric sleeve was the most common procedure (485, 73.3%). A substantial number were satisfied with the surgery results (293, 44.3% strongly agree), and many found the experience attractive (220, 33.2%). However, a notable portion suffered from excess or saggy skin (311, 47.0%).

**Table 2:** Features of the Participants Related to Bariatric Surgery

		Frequency (n=662)	Percent
Ever had bariatric surgery	No	85	12.8
	Yes	558	84.3
Weight of the patients	Before surgery (mean, SD)	121.1 (27.1)	
	After surgery (mean, SD)	84.7 (22.5)	
	p-value	< 0.001	
Type of bariatric surgery	Gastric sleeve	485	73.3
	Gastric balloon	34	5.1
	Gastric bypass	29	4.4
	Stomach belt	8	1.2
	Pancreaticobiliary diversion	2	0.3
Satisfied with the result of surgery	Strongly agree	293	44.3
	Ok	194	29.3
	Neutral	52	7.9
	Not agree	13	2
	Strongly disagree	6	0.9
Rate of experience after obesity surgery	Attractive	220	33.2
	Unattractive	20	3.0
	Neutral	153	23.1
	Very attractive	158	23.9
	Very unattractive	7	1.1
Suffer from excess or saggy skin	No	247	37.3
	Yes	311	47.0

Table 3 presents difficulties faced by participants due to saggy skin after bariatric surgery. Approximately 18.9% of participants reported experiencing "rashes/itching due to excess skin" several days a week. A significant 17.5% encountered challenges in "running/walking quickly because of excess skin" for several days each week. Emotional difficulties such as "lack of interest/pleasure in activities" (133, 20.1%) and "feeling frustrated/depressed/hopeless" (123, 18.6%) were prevalent over multiple days a week. Many participants faced issues with clothing choices, with "difficulty finding suitable clothes due to excess skin" reported by 19.5%. Additionally, a noteworthy proportion (102, 15.4%) experienced hindrances in their daily life due to excess skin on several days a week.

**Table 3:** Difficulties Faced by the Participants due to Saggy Skin After Bariatric Surgery

		Never	Almost everyday	Several days a week	More than half of a week
Little interest/pleasure in doing things	N	89	46	133	43
	%	13.4	6.9	20.1	6.5
Feel frustrated/depressed/hopeless	N	101	44	123	43
	%	15.3	6.6	18.6	6.5
Excess skin causes rashes/itching	N	145	21	108	37
	%	21.9	3.2	16.3	5.6
Excess skin makes it difficult to run/walk quickly	N	130	24	116	41
	%	19.6	3.6	17.5	6.2
Finding the right clothes is difficult for me due to extra skin	N	83	40	129	59
	%	12.5	6.0	19.5	8.9
My excess skin hinders me in everyday life	N	135	30	102	44
	%	20.4	4.5	15.4	6.6

Table 4 shows insights into participants who underwent body contouring or body lift surgery after bariatric surgery. Among the 662 participants, 8.3% had body contouring surgery. The types of surgeries included body lift (13, 23.6%), liposuction (19, 34.5%), and tummy tuck (26, 47.2%). Most procedures occurred greater than one year after bariatric surgery (31, 56.4%). A significant portion considered additional procedures (31, 56.4%), while 43.6% did not.

**Table 4:** Features of the Participants Who Had Body Contouring Surgery

		Frequency (n=55)	Percent
Ever had body contouring or body lift surgery (n=662)	Yes	55	8.3
Type of body contouring surgery	Body lift	13	23.6
	Liposuction	19	34.5
	Tummy tuck	26	47.2
	Other	5	9.0
Time of procedure	Immediately after bariatric surgery	5	9.0
	<1 year after bariatric surgery	19	34.5
	>1 year after bariatric surgery	31	56.4
Consider another procedure besides the previous one	No	24	43.6
	Yes	31	56.4

**Figure 1:**
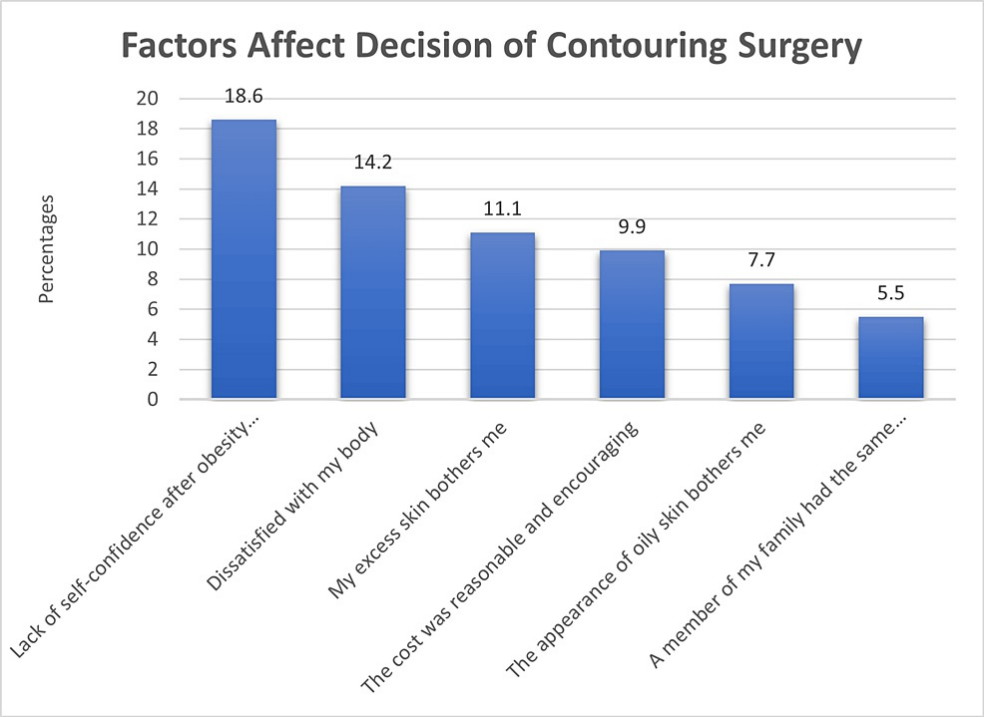
Factors Affecting the Decision of Contouring Surgery

Figure 1 shows the factors influencing the decision to undergo contouring surgery. The most significant factors were a lack of self-confidence (18.6%), body dissatisfaction (14.2%), and discomfort with excess skin (11.1%). A reasonable cost (9.9%) concerns about oily skin (7.7%), and family members' experiences (5.5%) also played roles in the decision-making process.

Table 5 shows the factors and barriers related to body-contouring surgery in patients who previously underwent bariatric surgery. Among 213 participants, 32.2% considered body-contouring surgery to improve physical appearance. The timing for considering surgery varied, with 52.5% contemplating it less than a year after bariatric surgery. Barriers included cost (171, 80.2%), fear of a second surgery (97, 45.6%), concerns about side effects (90, 42.2%), social factors (69, 32.3%), and busy surgery schedules (32, 15.0%). A notable portion (58, 27.2%) attempted to reduce costs by visiting other surgeons.

**Table 5:** Different Factors or Barriers to Contouring Surgery in Patients Who Have Undergone Bariatric Surgery

		Frequency (n=213)	Percent
Have you considered undergoing body-contouring surgery?	Yes	213	32.2
Undergoing body-contouring surgery will improve physical appearance.	No	4	1.8
	Yes	209	98.1
At what time following factors prevent you from undergoing body-contouring surgery?	Right after my bariatric surgery	47	22.1
	< 1 year after bariatric surgery	54	25.3
	< 1 year after bariatric surgery	112	52.5
Barriers to contouring surgery in patients who have undergone bariatric surgery.			
Not undergoing body-contouring surgery due to its cost.	No	42	19.7
	Yes	171	80.2
If yes, did you try to visit other surgeons to decrease the cost?	No	110	51.6
	Yes	58	27.2
Not undergoing body-contouring surgery due to fear of 2^nd^ surgery.	No	116	54.4
	Yes	97	45.6
Not undergoing body-contouring surgery due to fear of its side effects.	No	123	57.7
	Yes	90	42.2
Social factors like people's judgment about cosmetic surgery affect your decision about body-contouring surgery.	No	144	67.6
	Yes	69	32.3
Not undergoing body-contouring surgery due to busy appointments for surgery.	No	178	83.5
	Yes	32	15.0

Table 6 shows the sociodemographic factors and their significance in relation to the consideration of body-contouring surgery among patients who previously underwent bariatric surgery. Notably, females (143, 67.1%) were more likely than males (70, 32.9%) to consider it, with a significant p-value of 0.003. Similarly, nationality played a significant role, with Saudis (182, 85.4%) more inclined than non-Saudis (31, 14.6%) to consider surgery (p-value < 0.001). Occupation also showed significance, as employed (105, 49.3%) were more likely to consider surgery than others (p = 0.038).

**Table 6:** Different Sociodemographic Factors or Barriers to Contouring Surgery in Patients Who Have Undergone Bariatric Surgery BMI: Body mass index

			Thought About Undergoing Body Contouring Surgery		Sig. Value
			No	Yes	
Gender	Female	N	157	143	0.003
		%	53.8%	67.1%	
	Male	N	135	70	
		%	46.2%	32.9%	
Age	19-29 Years	N	138	84	0.133
		%	47.4%	39.4%	
	30-39 Years	N	76	72	
		%	26.1%	33.8%	
	40-49 Years	N	62	41	
		%	21.3%	19.2%	
	50-59 Years	N	15	15	
		%	5.2%	7.0%	
	60 or Greater	N	0	1	
		%	0.0%	0.5%	
Nationality	Non- Saudi	N	17	31	<0.001
		%	5.8%	14.6%	
	Saudi	N	275	182	
		%	94.2%	85.4%	
Marital Status	Single	N	129	78	0.249
		%	44.2%	36.6%	
	Married	N	148	119	
		%	50.7%	55.9%	
	Divorced	N	12	11	
		%	4.1%	5.2%	
	Widowed	N	3	5	
		%	1.0%	2.3%	
Education	Basic Education (to Secondary Level)	N	64	52	0.441
		%	21.9%	24.4%	
	University Level	N	212	154	
		%	72.6%	72.3%	
	Non-University	N	16	7	
		%	5.5%	3.3%	
Occupation	Student	N	49	41	0.038
		%	16.8%	19.2%	
	Employed	N	159	105	
		%	54.5%	49.3%	
	Unemployed	N	43	20	
		%	14.7%	9.4%	
	Housewife	N	41	47	
		%	14.0%	22.1%	
Monthly Income	< 2500 SAR	N	107	85	0.511
		%	36.6%	39.9%	
	2500-4999 SAR	N	54	39	
		%	18.5%	18.3%	
	5000-9999 SAR	N	60	48	
		%	20.5%	22.5%	
	10000-19999 SAR	N	54	27	
		%	18.5%	12.7%	
	> 20,000 SAR	N	17	14	
		%	5.8%	6.6%	
BMI	Underweight	N	1	1	0.144
		%	0.4%	0.5%	
	Normal	N	43	38	
		%	15.1%	18.4%	
	Overweight	N	76	72	
		%	26.8%	34.8%	
	Class 1 Obese	N	55	39	
		%	19.4%	18.8%	
	Class 2 Obese	N	43	21	
		%	15.1%	10.1%	
	Class 3 Obese	N	66	36	
		%	23.2%	17.4%	

## Discussion

Obesity rates have tripled globally since 1975, with a 35.6% prevalence in Saudi Arabia. Bariatric surgery, a common treatment, often leads to excess skin. Despite its benefits, few patients choose body-contouring surgery. Our study aims to identify barriers, including socioeconomic and psychological factors. Our findings on body-contouring surgery barriers after bariatric surgery offer valuable insights. We discuss results in relation to existing literature and their implications for clinical practice and future research.

Our study found a predominantly female (58.3%) participant group, which is in line with previous research showing women's higher interest in post-weight loss body contouring as women reported significantly more problems, discomfort, and amount of excess skin (p < 0.05) than men [14]. Most participants (44.3%) fell within the 19-29 age group, consistent with the trend of bariatric surgeries being common among younger adults [15]. The high representation of Saudi nationals (91.5%) aligns with the country's demographic profile.

Females were notably more inclined to consider body-contouring surgery (67.1%) than males (32.9%), aligning with prior research highlighting a higher prevalence of such procedures among women. This underscores the well-established link between gender, body image, and the desire for body-contouring surgery, as shown by Giordano et al. in which 69.4% of females have a desire to undergo post-bariatric body-contouring surgery [16].

Non-Saudi nationals (14.6%) were less inclined to consider body-contouring surgery than Saudi nationals (85.4%, p < 0.001). Cultural norms, healthcare access, and socioeconomic status likely contribute to this distinction.

Occupation influenced the consideration of body-contouring surgery, with employees, students, and housewives showing higher interest, possibly due to flexible schedules. This underscores the importance of considering diverse patient motivations.

Our study revealed that 84.3% of participants had undergone bariatric surgery, aligning with Saudi Arabia's high prevalence of these procedures for increasing obesity prevalence with 41% in men and 78% in women by 2022 [17]. The substantial weight reduction, from 121.1 kg to 84.7 kg, highlights bariatric surgery's effectiveness in achieving significant weight loss, consistent with prior research on various bariatric procedures [18].

Gastric sleeve, representing 73.3% of bariatric procedures, aligns with the global popularity due to its effectiveness and lower complications [19]. Participant satisfaction (44.3% strongly agree) underscores bariatric surgery's positive impact, improving weight, health, and overall quality of life, consistent with prior research where 70-90% are generally satisfied with bariatric surgeries [20].

A significant portion (47.0%) of participants experienced excess or saggy skin after weight loss, a common issue due to the skin's struggle to adapt to reduced body size [21]. Excess skin causes physical discomfort, skin-related problems, and emotional distress, motivating consideration of body-contouring surgery [11].

Several key factors, identified by our study, influence the decision to undergo body-contouring surgery. Notably, a lack of self-confidence was the most significant motivator (18.6%), followed by body dissatisfaction (14.2%) and discomfort with excess skin (11.1%). These findings align with prior research emphasizing body image concerns as primary drivers for body-contouring surgery [22].

Cost emerged as a significant factor for some participants (9.9%), underscoring its impact as a barrier to accessing these procedures. While not ranking as high as body image-related factors, it may be a limiting factor [23]. Thus, addressing financial considerations is crucial to enhance accessibility.

The influence of family members' experiences (5.5%) suggests that social support and shared family dynamics may shape individuals' choices in pursuing body-contouring surgery. The results align with other research highlighting social networks' impact on healthcare decisions [24].

There are several barriers to body-contouring surgery in post-bariatric surgery patients. Cost was a prominent hindrance, with 80.2% citing it as a deterrent, aligning with prior research highlighting cost as a significant barrier. Concerns about a second surgery (45.6%) and potential side effects (42.2%) were substantial barriers, possibly due to the complexity and associated risks. Societal judgments about cosmetic surgery (32.3%) contributed to hesitancy, emphasizing the importance of reducing stigma. Additionally, busy surgical schedules (15.0%) underscored the need for improved access and efficient scheduling [25].

Limitations

This study has some limitations, including its cross-sectional design, which limits the ability to establish causation. Additionally, the study was conducted in a specific region (Saudi Arabia), and the findings may not be generalizable to other populations. Further research with larger and more diverse samples is needed to validate these findings and explore additional factors that may influence the decision-making process for body-contouring surgery.

## Conclusions

Our study sheds light on the considerations, barriers, and sociodemographic factors associated with body contouring surgery among individuals who have previously undergone bariatric surgery in Saudi Arabia. The findings underscore the multifaceted nature of these decisions and the need for a patient-centered approach to care. Addressing barriers and providing comprehensive support can help individuals make informed choices regarding body contouring surgery and improve their overall well-being.
